# Anti-inflammatory effectiveness of *Peperomia pellucida* (L.) Kunth in rats induced with periodontitis

**DOI:** 10.1016/j.bbrep.2024.101856

**Published:** 2024-10-26

**Authors:** Dewi Lidya Ichwana Nasution, Sri Tjahajawati, Ratna Indriyanti, Amaliya

**Affiliations:** a Doctoral Program of Dentistry, Faculty of Dentistry, Padjadjaran University, Indonesia; b Department of Periodontic, Faculty of Dentistry Jenderal Achmad Yani University, Indonesia; c Departement of Oral Biology, Faculty of Dentistry, Padjadjaran University, Indonesia; d Departement of Pediatric Dentistry Faculty of Dentistry, Padjadjaran University, Indonesia; e Department of Periodontic, Faculty of Dentistry, Padjadjaran University, Indonesia

**Keywords:** Anti-Inflammatory, Pocket probing depth, Peperomia pellucida (L.) kunth extract, Periodontitis

## Abstract

**Background:**

Periodontitis, marked by deep periodontal pocket depth (PPD), facilitates bacterial colonization and inflammation, necessitating adjunctive therapies. Although 0.2 % chlorhexidine (CHX) mouthwash is effective, its side effects have led to the search for alternative treatments. Peperomia pellucida (L.) Kunth, known as Pepper Elder, is a traditional medicinal plant with potential as an adjunctive herbal therapy for periodontitis.

**Objective:**

This study aimed to investigate the anti-inflammatory efficacy of Peperomia pellucida extract in rats with induced periodontitis.

**Methods:**

A post-test control group design was used in this laboratory experimental study. Four groups of Wistar strain *Rattus norvegicus* rats were utilized: a Pristine group (without periodontitis), a negative control group (induced periodontitis only), a positive control group (induced periodontitis and administered 0.2 % CHX), and an experimental group (induced periodontitis and administered 2.5 μL of Pepper Elder extract). Each treatment group received daily administration for one week. PPD measurements were taken on days 0, 3, 5, and 7. Blood serum was collected on day 7 for ELISA to measure IL-1β, TNF-α, IL-10, and IL-13 levels. Statistical analysis was performed using the Kruskal-Wallis test with a post hoc LSD and Mann-Whitney test.

**Results:**

The extract-treated rats showed a decrease in PPD, with significant differences between the extract group and the negative control group (p < 0.05). TNF-α levels in the extract group differed significantly from the negative control group (p < 0.05) but not from the Pristine and positive control groups. IL-1β levels differed significantly only from the negative control group. IL-10 levels were significantly different from both the Pristine and negative control groups, while IL-13 levels differed significantly only from the negative control group.

**Conclusion:**

Peperomia pellucida (L.) Kunth extract exhibits anti-inflammatory effects in rats with induced periodontitis.

## Highlights

• *Peperomia pellucida* extract reduces inflammatory cytokines in rat models.
• Significant decrease in TNF-α and IL-1β levels post-treatment observed.
• Improved anti-inflammatory response with increased IL-10 and IL-13.
• Natural extract shown as a potential therapy for periodontal inflammation.
• Study supports the use of herbal remedies in managing oral diseases.

## 1 Introduction

Periodontitis is a chronic inflammatory disease influenced by multiple factors and is linked to the buildup of dental plaque. This condition is marked by the progressive damage of periodontal tissues, which include the periodontal ligament, cementum, and alveolar bone. The disease progresses through a complex and dynamic interaction between specific bacterial pathogens and a harmful immune response [[1], [2], [3], [4]]. The host's local inflammatory response, aimed at inhibiting microbial growth, can also act as a nutrient source for subgingival plaque bacteria [5,6].

In chronic periodontitis, cytokines are crucial regulators of the inflammatory processes that contribute to disease progression. Pro-inflammatory cytokines, including IL-1β and TNF-α, drive the inflammatory response by promoting the production of tissue-degrading enzymes such as matrix metalloproteinases (MMPs) and enhancing osteoclast activity, resulting in alveolar bone loss and destruction of periodontal support structures. Elevated levels of these cytokines correlate with increased tissue damage and disease severity. In contrast, anti-inflammatory cytokines like IL-10 and IL-13 play a protective role by moderating the immune response, reducing the production of pro-inflammatory cytokines, and supporting the healing and regeneration of periodontal tissues. The imbalance between pro- and anti-inflammatory cytokines is a critical factor influencing the severity of chronic periodontitis. A deeper understanding of these cytokines in the pathogenesis of periodontitis may lead to therapeutic strategies that target cytokine regulation to control inflammation and protect against further tissue damage [[7], [8], [9], [10]].

The primary objective of periodontal treatment is to address the inflammatory process, halt disease progression, and prevent tooth loss. Traditionally, periodontal therapy has focused on disrupting and eliminating biofilm pathogens. The pathophysiology of periodontitis involves multiple mechanisms, requiring the identification of local and systemic risk factors that negatively affect the quantity and quality of plaque bacteria and the immune response. Mechanical debridement of tooth and root surfaces through scaling and root planing (SRP) is considered the gold standard for periodontal therapy [5,11]. Numerous studies have explored the adjunctive use of antibiotics in the treatment of periodontitis. While this approach is generally more affordable, easier to administer, and relatively safe for short-term use, it has several limitations. These include potential side effects, the risk of allergic reactions, bacterial resistance, drug toxicity, and the need for higher doses to achieve the required concentrations in the gingival crevicular fluid at the target site [6,[12], [13], [14], [15]].

The direct application of antimicrobials or synthetic antiseptic solutions to the subgingival area can minimize fluid penetration into the tissues, thereby reducing systemic effects. However, this approach can still cause allergic reactions, and some antiseptics are not safe for use in pregnant and breastfeeding patients [16]. Therefore, there is a need for a natural substance with high safety and antibacterial properties to replace synthetic materials in periodontal treatment [17].

Herbal medicines and formulations consisting of plant elements have been recognized for their therapeutic benefits. Compared to chemical drugs, herbal products offer natural activity, a higher safety margin, and lower costs [17]. Numerous studies have shown that the addition of herbal medicines to SRP therapy in the treatment of periodontitis results in better periodontal tissue improvement compared to SRP alone. This has been evaluated through periodontal parameters such as reductions in the Gingival Index (GI), Plaque Index (PI), Bleeding Index, and Periodontal Probing Depth (PPD), along with significant improvements in tissue attachment levels [[17], [18], [19], [20], [21], [22], [23], [24]]. The addition of herbal components in periodontal treatment, whether applied locally or systemically, has been shown to significantly reduce the enzymatic activity of microorganisms, decrease the number of pathogenic bacteria in the oral cavity, and lower inflammatory markers [18,21].

Flavonoids and polyphenols, bioactive compounds present in various plant extracts, are known to influence cytokine production, which plays a key role in inflammatory processes. These compounds help regulate the immune response by reducing pro-inflammatory cytokines like IL-1β and TNF-α, and promoting anti-inflammatory cytokines such as IL-10 and IL-13. This regulation occurs through the inhibition of pathways such as NF-κB and MAPK. Consequently, the presence of these compounds in plant extracts could offer therapeutic benefits for managing periodontitis [25,26].

*Peperomia pellucida* (L.) Kunth, commonly known as Pepper Elder, is known to contain various bioactive compounds and exhibits recognized antibacterial, antioxidant, and anti-inflammatory properties. Several studies have explored its potential health benefits. However, there is currently no research that specifically addresses the effectiveness of *Peperomia pellucida* in promoting the healing of periodontal tissues affected by chronic inflammation or periodontitis, particularly in relation to its impact on reducing pro-inflammatory cytokines and enhancing anti-inflammatory cytokines, which are crucial in the healing process of chronic periodontitis.

## 2 Materials and methods

This study is a laboratory experimental research with a post-test control group design. Pepper Elder leaf extract (*Peperomia pellucida* (L.) Kunth) is the preparation to be tested.

### 2.1 Preparation of *Peperomia pellucida (L.) Kunth* extract

The Pepper Elder plants were obtained from the yard of the Masjid Peradaban Percikan Iman Arjasari, Bandung Regency, West Java, Indonesia. Leaf determination tests for Pepper Elder were conducted at the Biosystematics and Molecular Laboratory, Department of Biology, Faculty of Mathematics and Natural Sciences, Padjadjaran University, Bandung, West Java, Indonesia. The extract was prepared using the reflux method, which involves extracting using a solvent at its boiling point temperature, over a specific period, with a relatively constant solvent volume and the presence of a reflux condenser. One of the effective and commonly used polar solvents is 96 % ethanol. The fundamental principle of the reflux method is to use the solvent, then condense it back to liquid form and return it to the reaction vessel. The solvent remains present throughout the reaction, allowing the process to proceed effectively [27,28].

1 kg of selected Pepper Elder leaves were thoroughly washed and dried in an oven, then blended into powder. The powder and 96 % ethanol were placed into a round-bottom flask until all the powder was submerged. The flask was set up in a reflux apparatus connected to a condenser and heated for 1 h. Filtrate was obtained from the filtered sample using filter paper, and the solvent was evaporated to remove it using a rotary evaporator, resulting in Pepper Elder leaf extract, which was then diluted with dimethyl sulfoxide [27,28].

### 2.2 Phytochemistry of *Peperomia pellucida* leaf extract

Phytochemical screening of *Peperomia pellucida* (L.) Kunth reflux extracts was conducted to identify the presence of alkaloids, polyphenols, tannins, flavonoids, quinones, saponin, monoterpenoids, sesquiterpenoids, steroids, and triterpenoids [29].

### 2.3 Thin Layer Chromatography (TLC) test of flavonoid and polyphenol compounds

Thin Layer Chromatography (TLC) was used for the identification of flavonoids and polyphenols in *Peperomia pellucida* leaf extract [30]. Silica gel plates (Merck-silica gel 60 F_254_, 8x2 cm) were activated by heating. The sample was applied using a capillary tube, and the plate was placed in a chromatography chamber saturated with the mobile phase. Elution was performed up to 6 cm from the baseline. using a solvent ratio of n-hexane:ethyl acetate (8:12) for flavonoid identification [31] and chloroform:methanol (9:1) for polyphenol identification, based on previous research [32]. The plates were then removed from the chamber and dried at room temperature. After the TLC plates were dry, visualization was performed using chromatography with ammonia reagent for flavonoids and FeCl_3_ for polyphenols before and after color detection under visible light, UV light at 254 nm, and UV light at 366 nm. Rf values were calculated based on the distance traveled by the spots in relation to the solvent front using the standard formula for Rf:

$$\text{Rf}=\frac{Distance\;of\;centre\;of\;spot\;from\;starting\;point}{Distance\;of\;solvent\;front\;from\;starting\;point}$$

This calculation allows for the identification of specific compounds based on their movement on the TLC plate under the experimental conditions**.** [30].

### 2.4 Experimental animals

The research subjects are male Wistar strain *Rattus Norvegicus* rats obtained from the Animal Laboratory of the Faculty of Medicine, Jenderal Achmad Yani University, Cimahi, West Java, Indonesia, aged 8–12 weeks with a weight of 170–180 g, in good physical condition without abnormalities [33,34]. The sample size is determined using the Federer formula, and the minimum number of samples in each group is 5 rats. Rats that die during the adaptation period and periodontitis induction process, as well as rats with loose wire ligatures after 3 days of placement during the periodontitis induction process, are excluded from the study.

### 2.5 Inducing experimental periodontitis (EPD) in rats

The study was authorized by the local Ethics Committee of Faculty of Medicine, Jenderal Achmad Yani University, Cimahi, West Java, Indonesia No: 032/UH4.11/2023. The preparation for the research involved setting up the necessary equipment and protocols, as well as selecting research objects and subjects that met the inclusion criteria. Adaptation of the Wistar strain *Rattus norvegicus* rats was conducted for one week prior to the experimental period in rat cages measuring 40 cm x 60 cm x 60 cm in an animal laboratory with controlled conditions (AC set at 20°C–22 °C and humidity at 50–60 %) and a 12-h light-dark cycle. The rats were provided with ad libitum access to water and standard commercial rodent pellets. The care and use of all laboratory animals were conducted in accordance with the guidelines of the National Academy of Science [[33], [34], [35], [36]].

**Fig. 1:**
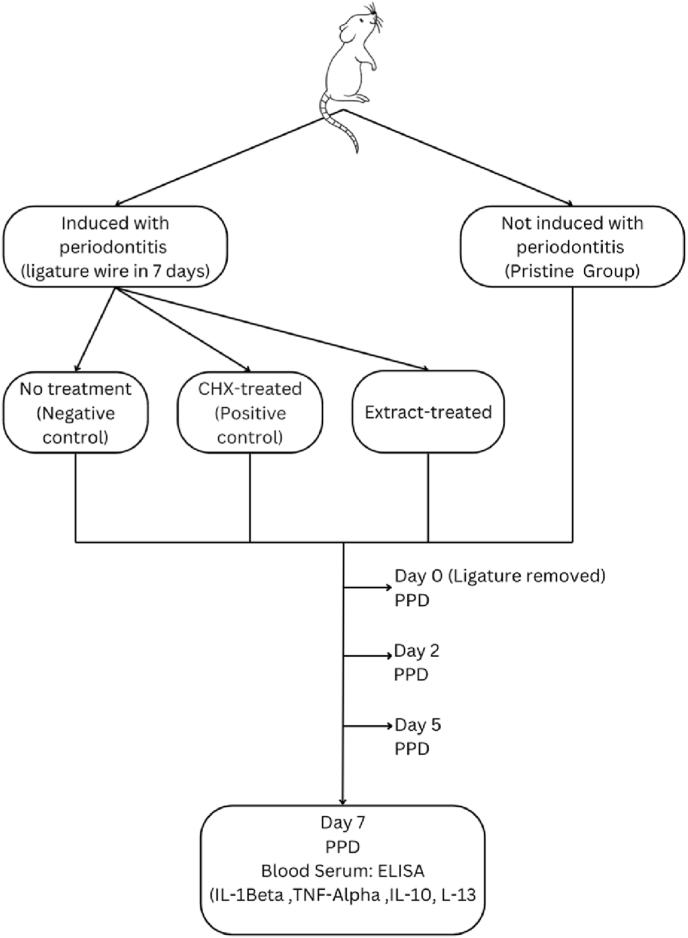
The stages of research on animal models.

Periodontitis induction was conducted on 3 groups: a negative control group, a positive control group treated with 0.2 % Chlorhexidine (CHX), and a group treated with 2,5 μL of Pepper Elder leaf extract (Fig. 1). The dosage of 2.5 μL of *Peperomia pellucida* was determined based on previous studies, which indicated that a concentration of 50 % exhibited strong antibacterial activity against *Porphyromonas gingivalis* and *Aggregatibacter actinomycetemcomitans*, both of which are the key bacteria involved in periodontitis [37,38]. This induction involved 2 dentoalveolar segments in each rat, the upper left and right incisors, as these locations are more easily accessible during manipulation. All procedures were performed after intraperitoneal anesthesia using ketamine HCl (40–80 mg/kg body weight) and xylazine (Xyla) (5–10 mg/kg body weight). The average duration of anesthesia for the rats was 1 h [33,34].

The ligature-induced periodontitis model is frequently employed to simulate periodontitis conditions in humans, especially for studying periodontal disease mechanisms and evaluating new therapeutic approaches. This model effectively replicates alveolar bone loss and periodontal soft tissue inflammation similar to clinical manifestations in humans. The placement of ligatures around the teeth promotes continuous plaque accumulation and inflammatory infiltration, leading to periodontal tissue destruction within approximately seven days. However, this model cannot fully replicate all aspects of human periodontitis due to differences in oral structure and microbiota composition between rats and humans. Despite these limitations, the ligature-induced model is considered valuable for investigating the pathogenesis of periodontitis, including the interaction between systemic diseases and periodontitis, as well as related immunological and bacteriological responses [39,40].

Periodontitis induction was performed on a sterile mouse dental bed with a dissecting lamp positioned on a flexible arm beside it. Induction was carried out once the rats were properly anesthetized, confirmed by checking for responsiveness through toe pinching. The induction procedure began by opening the interdental area between the upper left and right incisors using a 2.5 mm silk to facilitate ligature wire placement. Subsequently, 0.3 mm ligature wire was inserted in the cervical area of the teeth in an eight-shaped pattern using a probe and ligature wire tucker. The wire edges were twisted tightly using a needle holder to ensure adequacy and prevent easy detachment, then bent to avoid mucosal injury (Fig. 1). After ligature induction, dental plaque from chronic periodontitis patients (with probing pocket depth greater than 5 mm) was applied under the ligature wire using sterile paper point no. 40. Nicotine solution in physiological saline (0.001 mg/L) was then injected submucosally in the gingiva area, with injections administered once daily for a week using a disposable 1 cc syringe [34]. Nicotine administration can enhance susceptibility to periodontal destruction by inhibiting immune responses and promoting inflammatory processes, which is why it is used in conjunction with other methods like ligature placement. The concentration of nicotine and its delivery method are carefully chosen based on previous studies to ensure consistent systemic exposure and to replicate the impact of smoking-related periodontal disease. In these models, nicotine is shown to not only exacerbate periodontal damage but also delay the healing process after the removal of the ligature, reflecting the negative effects of smoking on periodontal health recovery [41].

**Fig. 2:**
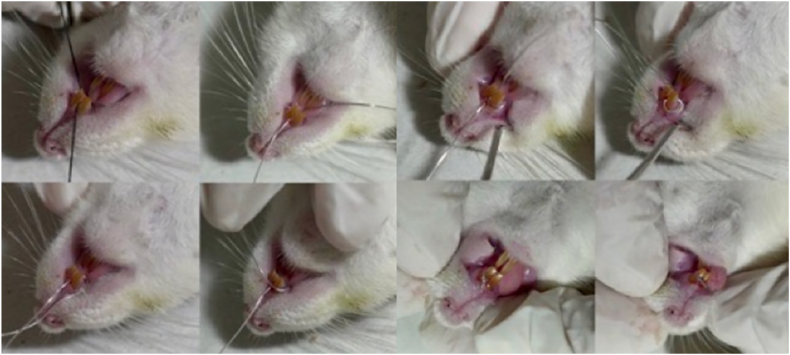
The induction of periodontitis by ligature placement in rats: (a) Opening interdental area with silk 2,5 mm. (b–f) 0.3 mm ligature wire was inserted into the cervical area of the teeth in an eight-shaped pattern using a probe and ligature wire tucker. (g–h) The wire ends were tightly twisted using a needle holder to ensure adequacy and prevent easy detachment."

The ligature wire was removed on the 7th day or once periodontitis was confirmed in the rats, indicated by changes in the color and consistency of the gingiva, gingival bleeding upon probing, formation of periodontal pockets (probing pocket depth greater than 1 mm), and tooth mobility (Fig. 2) [[34], [35], [36],^42^].

**Fig. 3:**
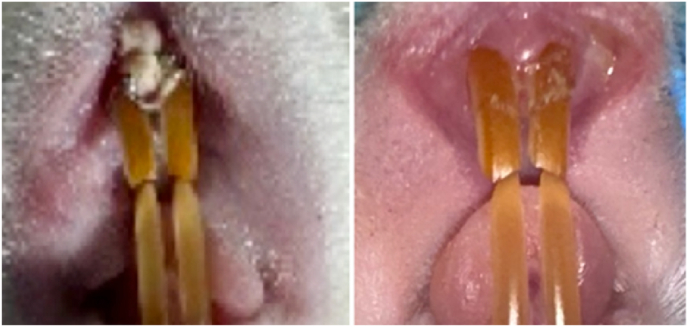
Before and 7 days after periodontitis induction.

### 2.6 Evaluation of probing pocket depth (PPD) in rats induced with periodontitis

Twenty-four male Wistar rats (*Rattus norvegicus*) were randomly divided into 4 groups and treated according to their groupings using a needle and a 1 cc disposable syringe at the test site:

1. Pristine Group: No periodontitis and no solution or treatment was administered.
2. Negative Control Group: Induced with periodontitis but not given any solution.
3. Positive Control Group:Induced with periodontitis and given 0.2 % CHX (Minosep), 0.05 ml topically around the teeth induced with periodontitis.
4. Extract Group: Induced with periodontitis and given 2.5 μL of Pepper Elder leaf extract, 0.05 ml topically around the teeth induced with periodontitis.

Each treatment group underwent daily treatment at noon for one week. After confirming periodontitis in the rats, periodontal pocket depth (PPD) was measured using a Periodontal Probe (UNC 15, Osung MND Co., Seoul, Korea). PPD measurements were performed by the same trained researcher, who was blinded to the treatment groups. The probe was carefully inserted into the periodontal pocket, ensuring that the probe remained parallel to the long axis of the tooth and in contact with the tooth surface.

The normal PPD value in rats is relatively smaller than in humans, typically less than 1 mm, due to anatomical differences. In this study, PPD was measured on the labial side of the right and left upper incisors, from the gingival margin to the base of the pocket. Prior to PPD measurement, the rats were anesthetized. PPD assessments were carried out on the same day as the ligature removal (day 0), as well as on days 3, 5, and 7 after periodontitis had been confirmed in the rats. The time points selected for PPD assessment in the rat periodontitis model were based on the progression and characteristics of periodontal inflammation and tissue damage observed in the model. These intervals allowed researchers to capture various stages of periodontal disease development and healing processes. By measuring PPD at different time points, such as day 0, 3, 5, and 7, it was possible to monitor the progression of inflammation and tissue response, providing a comprehensive understanding of disease dynamics and therapeutic efficacy [43]. PPD values for each group were then compared.

### 2.7 Blood serum collection

On the 7th day, the rats were anesthetized, and 10 ml of blood was collected from the inferior vena cava to obtain serum, which was used to measure the levels of pro-inflammatory cytokines TNF-α and IL-1β, as well as anti-inflammatory cytokines IL-10 and IL-13. The serum was allowed to clot for 10–20 min at room temperature, then centrifuged at 2000–3000 RPM for 20 min, and the supernatant was collected without any sediment. Day 7 is a crucial time point for observing changes in pro-inflammatory and anti-inflammatory cytokine levels, as this period typically represents the peak of the inflammatory process, with clear evidence of tissue damage. It is often considered the optimal time to measure significant cytokine fluctuations, reflecting the immune response to periodontal tissue destruction. This time point allows for a comprehensive assessment of the body's inflammatory and healing responses in experimental periodontitis models [43,44].

The rats were euthanized by decapitation following an overdose of ether administered intramuscularly, followed by cervical dislocation. All animals were anesthetized by intraperitoneal injection of a mixture (1 mL/kg) of Zoletil (Virbac) and Rompun (Bayer) in a 4:1 v/v ratio. Transcardial perfusion was performed with 0.1 M phosphate-buffered saline (PBS, pH 7.4) followed by 4 % paraformaldehyde in 0.1 M phosphate buffer (PB, pH 7.4). The maxillary tissue induced with periodontitis was then excised and immersed in 10 % paraformaldehyde in 0.1 M phosphate buffer (PB, pH 7.4) [45].

### 2.8 ELISA examination

Measurement of TNF-α, IL-1β, IL-10, and IL-13 levels was conducted using commercially available enzyme-linked immunosorbent assay (ELISA) kits (Medik Bio, Rat Tumor Necrosis Factor Α, TNF-A ELISA Kit Cat.No MDE0764Ra, Rat Interleukin 1 Beta, IL-1B ELISA Kit Cat.No MDBE0119Ra, Rat Interleukin 10, IL-10 ELISA Kit Cat.No MDE0108Ra, Rat Interleukin 13, IL-13 ELISA Kit Cat.No MDB0112Ra). The manufacturer's instructions were strictly followed during the testing process. All reagents, standard solutions, and samples were prepared and tested at room temperature.

The ELISA samples and reagents were added to each well and incubated for 1 h at 37 °C, then the plates were washed 5 times. Substrate solutions A and B were then added and incubated for 10 min at 37 °C. The stop solution was added until a color change occurred, and the optical density (OD) values were read within 10 min. The average OD results were calculated using computer-based curve fitting software, and the best-fit line was determined through regression analysis [[45], [46], [47]].

### 2.9 Statistical analysis

Data analysis was initiated with the Shapiro-Wilk test for normality and Levene's Test for homogeneity. If data were normally distributed and homogeneous, One-Way ANOVA was used; otherwise, the Kruskal-Wallis test was applied. Significant differences between groups, with and without *Peperomia pellucida* extract, were assessed using the Mann-Whitney test. Statistical analyses were conducted using SPSS software.

## 3 Results

The study was conducted at the Animal Laboratory of the Faculty of Medicine, Universitas Jenderal Achmad Yani. This research is a pure in vivo study designed as a post-test control group design. The study aimed to investigate the anti-inflammatory effects of Pepper Elder leaf extract, assessed through clinical examination of probing pocket depth (PPD) and levels of pro-inflammatory and anti-inflammatory cytokines in Wistar rats (*Rattus norvegicus*) induced with periodontitis.

### 3.1 Phytochemical screening

The phytochemical screening results of the maceration and reflux extracts of *Peperomia pellucida* show that both extracts contain all the tested compounds except for saponins, triterpenoids, and steroids, as shown in Table 1.

**Table 1:** Phytochemical screening of reflux extract of *Peperomia pellucida* (L.) Kunth.

Compound Class	Result
Alkaloid	+
Plyphenol	+
Tannin	+
Flavonoid	+
Quinone	+
Saponin	–
Monoterpenoids and Sesquiterpenoids	–
Steroid and Triterpenoids	+

### 3.2 Flavonoid and polyphenol identification

**Fig. 4:**
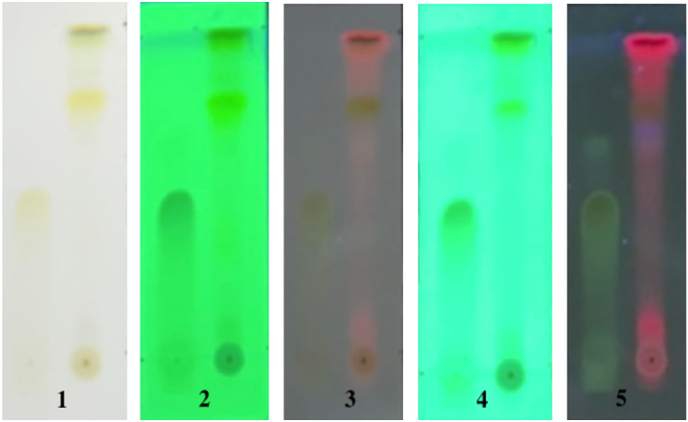
Motion phase, n-Hexane: Ethyl acetate (8 : 12) Flavonoid. Figure Descriptions: 1. TLC plate visual after spotting 2. TLC plate under UV light at 254 nm before spot visualizing spray 3. TLC plate under UV light at 356 nm before spot visualizing spray 4. TLC plate under UV light at 254 nm after spot visualizing spray with ammonia vapor 5. TLC plate under UV light at 356 nm after spot visualizing spray with ammonia vapor - First spot: Quercetin standard - Second spot: Extract sample.

**Fig. 5:**
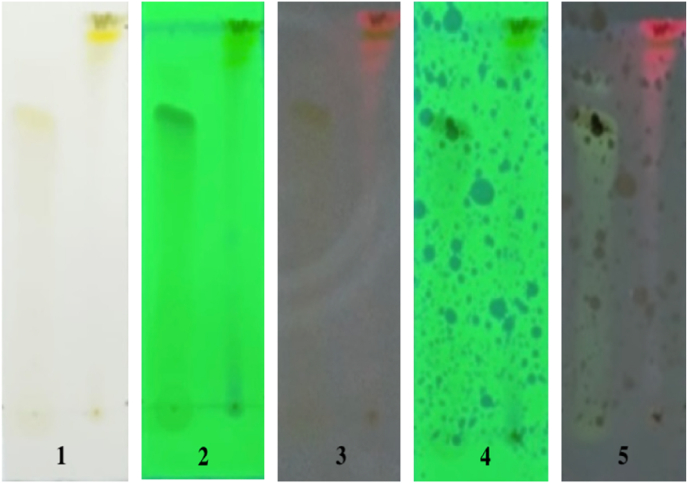
Motion Phase , chloroform: methanol (9 : 1) Polyphenol. Figure Descriptions: 1. TLC plate visual after spotting 2. TLC plate under UV light at 254 nm before spot visualizing spray 3. TLC plate under UV light at 356 nm before spot visualizing spray 4. TLC plate under UV light at 254 nm after spot visualizing spray with 10 % FeCl_3_ 5. TLC plate under UV light at 356 nm after spot visualizing spray with 10 % FeCl_3_ - First spot: Quercetin standard - Second spot: Extract sample.

**Fig. 6:**
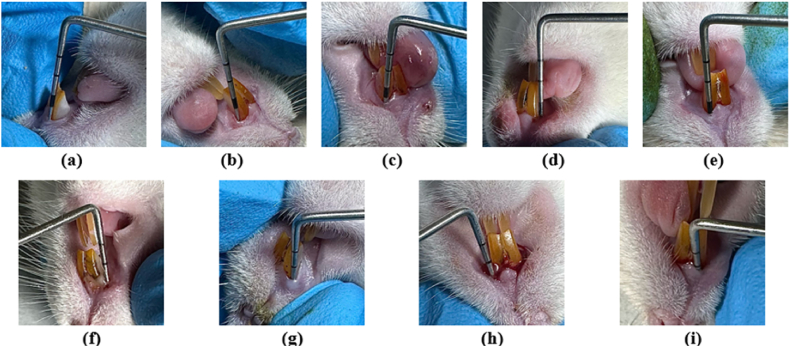
Result of PPD measurement. (a) 0,5 mm. (b) 1 mm. (c) 2 mm. (d) 3 mm. (e) 4 mm. (f) 5 mm. (g) 6 mm. (h) 7 mm. (i) 8 mm. Cited from: Personal documentation.

In the Thin Layer Chromatography (TLC) test for flavonoid identification, the eluent used was n-Hexane: Ethyl acetate (8:12) (see Fig. 3). The polyphenol test was conducted with the eluent chloroform: methanol (9:1). The results of the spotting of the extract on the TLC plate can be seen in Fig. 4, Fig. 5. The results indicated the presence of flavonoid and polyphenol compounds in the chromatogram pattern, as evidenced by the appearance of purple spots after being sprayed with ammonia and FeCl_3_ (see Fig. 6).

Rf Value

$$\text{Rf}=\frac{The\;migration\;distance\;covered\;by\;the\;sample}{The\;migration\;distance\;covered\;by\;the\;mobile\;phase}$$

$$\begin{array}{cc} \text{Reference} & \text{Pepper}\;\text{Elder}\;\text{Extract}\\ \text{Rf}=\frac{3\;cm}{4,5\;cm\;}=0,66 & \text{Rf}=\frac{3\;cm}{4,5\;cm\;}=0,66 \end{array}$$

∗ Showing positive results with the appearance of purple color on the extract, and the Rf value is the same as the quercetin standard.

Rf Value

$$\text{Rf}=\frac{The\;migration\;distance\;covered\;by\;the\;sample}{The\;migration\;distance\;covered\;by\;the\;mobile\;phase}$$

$$\begin{array}{cc} \text{Reference} & \text{Pepper}\;\text{Elder}\;\text{extract}\\ \text{Rf}=\frac{4\;cm}{4,5\;cm\;}=0,88 & \text{Rf}=\frac{4\;cm}{4,5\;cm\;}=0,88 \end{array}$$

∗ Showing positive results with the appearance of purple color on the extract, and the Rf value is the same as the quercetin standard.

### 3.3 Analysis of clinical measurement of PPD (probing pocket depth)

The Wistar rats (*Rattus norvegicus*) were randomly divided into four groups: the Pristine group, which was not induced with periodontitis and received no treatment; the negative control group, which was induced with periodontitis but received no treatment; the positive control group, which was induced with periodontitis and treated with 0.2 % CHX; and the experimental group, which was induced with periodontitis and treated with Pepper Elder leaf extract. Each group consisted of five rats. PPD measurements and recordings were conducted on the day the ligature wire was removed (day 0), day 3, day 5, and day 7 on the right and left maxillary incisors, as illustrated in Fig. 1.

Based on Table 2, in the Pristine group, there was no change in PPD from day 0 to day 7. In the negative control group, there was a decrease in the average PPD from 5.17 mm to 5.83 mm on day 0–3.5 mm and 4.17 mm on day 7. In the positive control group using 0.2 % CHX, there was a decrease in the average PPD from 3.83 mm to 4.5 mm on day 0–1.33 mm and 1.5 mm after treatment for 7 days. In the group treated with 50 % Pepper Elder extract, there was a decrease in the average PPD from 4.17 mm on day 0–1.33 mm and 1.08 mm after treatment for 7 days (see Table 3).

**Table 2:** Descriptive analysis of PPD (Probing Pocket Depth) on day 0, day 3, day 5, and day 7.

Group	Right incisor		Left incisor	
	Mean (St.Dev)	Min-Max	Mean (St.Dev)	Min-Max
**Day 0**				
*Pristine* group	0,67 (0,26)	0,5–1,0	0,67 (0,26)	0,5–1,0
Negative control group	5,17 (1,33)	3,0–7,0	5,83 (1,47)	4,0–8,0
Positive control *CHX* 0,2 % group	3,83 (0,75)	3,0–5,0	4,50 (1,38)	3,0–7,0
*P.pellucida* extract group	4,17 (2,04)	0,0–5,0	4,17 (2,71)	0,0–8,0

**Day 3**				
*Pristine* group	0,67 (0,26)	0,5–1,0	0,67 (0,26)	0,5–1,0
Negative control group	5,17 (1,17)	4,0–7,0	5,67 (1,03)	4,0–7,0
Positive control *CHX* 0,2 % group	2,83 (2,04)	0,0–6,0	3,33 (2,34)	0,0–7,0
*P.pellucida* extract group	2,50 (1,38)	0,0–4,0	2,33 (1,97)	0,0–5,0

**Day 5**				
*Pristine* group	0,67 (0,26)	0,5–1,0	0,67 (0,26)	0,5–1,0
Negative control group	4,50 (1,76)	3,0–8,0	4,33 (1,51)	3,0–7,0
Positive control *CHX* 0,2 % group	2,17 (2,04)	0,0–6,0	2,00 (1,10)	0,0–3,0
*P.pellucida* extract group	1,67 (1,03)	0,0–3,0	1,50 (1,05)	0,0–3,0

**Day 7**				
*Pristine* group	0,67 (0,26)	0,5–1,0	0,67 (0,26)	0,5–1,0
Negative control group	3,50 (2,35)	2,0–8,0	4,17 (1,72)	2,0–7,0
Positive control *CHX* 0,2 % group	1,33 (0,82)	0,0–2,0	1,50 (1,05)	0,0–3,0
*P.pellucida* extract group	1,33 (1,03)	0,0–3,0	1,08 (0,80)	0,0–2,0

**Table 3:** Paired comparison test of Probing Pocket Depth (PPD) between the Pepper Elder extract group and the Pristine group, the negative control group and the positive control group.

Day		*P.pellucida* extract group Vs *Pristine*	*P.pellucida* extract group Vs No treatment (negative control)	*P.pellucida* extract group Vs CHX group (positive control)
Day 0	Right incisor	0,042^a^	0,295	0,101
	Left incisor	0,050	0,195	0,803
Day 3	Right incisor	0,048^a^	0,006^a^	0,934
	Left incisor	0,097	0,009^a^	0,517
Day 5	Right incisor	0,069	0,004^a^	0,932
	Left incisor	0,096	0,006^a^	0,356
Day 7	Right incisor	0,128	0,032^a^	0,865
	Left incisor	0,309	0,006^a^	0,454

a *Mann Whitney*, p value < 0,05 (Significant).

Based on the paired comparison tests, significant differences were found between the 2,5 μL of Pepper Elder extract group and the Pristine group on certain days. However, these differences were not consistently observed across all experimental stages. Similarly, comparisons between Pepper Elder extract group and the negative control group showed significant differences on some days of the experiment, while on other days, the differences were not significant. The comparison test results also indicate that there were no significant differences between the Pepper Elder extract group and the positive control CHX 0.2 % group on most days of the experiment.

### 3.4 Analysis of the anti-inflammatory evaluation of IL-1β, TNF-α, IL-10, and IL-13 levels

On day 7 after periodontitis induction and treatment administration, blood was drawn from the inferior vena cava, totaling 10 mL, to obtain blood serum for determining the levels of proinflammatory cytokines TNF-α, IL-1β, and anti-inflammatory cytokines IL-10, IL-13 through ELISA examination.

In Table 4, the mean values of proinflammatory cytokines TNF-α and IL-1β were highest in the group of rats induced with periodontitis but not given any treatment, i.e., the negative control, with values of 135.850 ± 21.746 pg/mL for TNF-α and 6.328 ± 1.518 pg/mL for IL-1β. The highest mean value of the anti-inflammatory cytokine IL-10 was observed in the positive control group, which consisted of rats induced with periodontitis and treated with 0.2 % CHX, measuring 530.941 ± 193.601 pg/mL. The highest IL-13 mean value was found in the experimental group, comprising rats induced with periodontitis and treated with Chinese betel leaf extract, measuring 155.209 ± 7.555 pg/mL (see Table 5).

**Table 4:** Descriptive analysis of IL-1β, TNF-α, IL-10, and IL-13 levels on day 7.

Variable	Group	Mean (pg/mL)	Standard Deviation	Min-Max
TNF- α	*Pristine* group	93,946	8426	85,33–105,1
	Negative control group	135,850	21,746	105,90–164,69
	Positive control group	131,088	30,046	94,11–157,68
	*P. pellucida* group	87,304	41,029	36,22–149,66
IL-1β	*Pristine* group	2119	1116	0,71–3,48
	Negative control group	6328	1518	4,48–8,47
	Positive control group	0,755	0,378	0,42–1,29
	*P. pellucida* group	0,772	0,168	0,58–0,97
IL-10	*Pristine* group	162,823	7209	155,29–172,35
	Negative control group	185,882	72,814	145,29–315,29
	Positive control group	530,941	193,601	305,88–837,65
	*P. pellucida* group	458,353	142,029	295.88–658.82
IL-13	*Pristine* group	129,750	32,876	88,26–169,75
	Negative control group	48,128	13,458	32,03–64,97
	Positive control group	153,774	17,840	136,04–178,37
	*P. pellucida* group	155,209	7555	146,35–164,16

**Table 5:** Paired comparison tests on the anti-inflammatory levels of TNF-α, IL-1β, IL-10, and IL-13 between the Pepper Elder extract group and the Pristine group, negative and positive controls groups.

Antibody	Extract group Vs *Pristine* group	Extract group Vs Negative control group	Extract group Vs Positive control group
TNF- α	0.674	**0.005**^a^	**0.010**^a^
IL-1β	0.056	**0.008**^a^	1.000
IL-10	**0.008**^a^	**0.016**^a^	0.548
IL-13	0.222	**0.008**^a^	1.000

a Numeric data values were tested using the LSD test if the data followed a normal distribution, with the Mann-Whitney test used as an alternative if the data were not normally distributed and the variances were not homogeneous. A p-value of less than 0.05 was considered significant.

For the normality test, numerical data were assessed using the Shapiro-Wilk test due to the data size being <50, including TNF-α, IL-1β, IL-10, and IL-13. The normality test results indicated that the p-values for TNF-α, IL-1β, and IL-13 in all groups were greater than 0.05 (p > 0.05), indicating a normal distribution of data. Additionally, the normality test results showed that the p-values for the IL-10 variable in the Pristine group, experimental group, and positive control group were greater than 0.05 (p > 0.05), indicating a normal distribution of data. However, the p-value for the IL-10 variable in the negative control group was less than 0.05 (p < 0.05), indicating a non-normal distribution of data.

For the homogeneity test of the numerical data, the Levene Test was employed. The results of the homogeneity test indicated that the p-values for the IL-1β, IL-10, and IL-13 variables were less than 0.05 (p < 0.05), indicating non-homogeneous data. Conversely, the p-value for the TNF-α variable was greater than 0.05 (p > 0.05), indicating homogeneous data.

Based on the paired comparison tests, the results show that the TNF-α levels in the Pepper Elder extract group did not differ significantly from the Pristine group but differed significantly from the negative control group, which consisted of rats induced with periodontitis without treatment (p < 0.05). The TNF-α levels also differed significantly from the positive control group, which consisted of rats induced with periodontitis and treated with 0.2 % CHX (p < 0.05). For IL-1β levels, the comparison between the Pepper Elder extract group and the Pristine group, as well as the group of rats induced with periodontitis and treated with 0.2 % CHX, did not show significant differences (p > 0.05). However, there was a significant difference when compared to the group of rats induced with periodontitis without treatment, or the negative control group (p < 0.05).

For IL-10 levels, the paired comparison test results show that the Pepper Elder extract group differs significantly from the Pristine group and the negative control group (p < 0.05) but does not differ significantly from the positive control group, which consisted of rats induced with periodontitis and treated with 0.2 % CHX (p > 0.05). Similarly, for IL-13 levels, the Pepper Elder extract group does not differ significantly from the Pristine group and the positive control group (p > 0.05) but differs significantly from the negative control group, which consisted of rats induced with periodontitis without treatment (p < 0.05).

## 4 Discussion

Rats are widely utilized in periodontal research due to their cost-effectiveness, ease of handling, and biological relevance for modeling periodontal disease [34,40,48]. Three primary methods are used for inducing periodontitis in research: ligature placement, lipopolysaccharide (LPS) injection, and bacterial inoculation. Ligature placement, in particular, is an effective method as it promotes the accumulation of plaque, soft deposits, and calculus, creating an environment conducive to bacterial colonization, thereby leading to the rapid onset of periodontitis. This method is known to produce histopathological features that closely resemble human periodontitis [48,49]. Previous studies have demonstrated that maximum alveolar bone loss (ABL) occurs between days 7 and 10 after the placement of nylon ligatures, with the extent of ABL decreasing by day 14. However, one limitation of this approach is its focus on local effects without addressing the infectious nature of periodontitis [34].

The accumulation of plaque over time leads to an inflammatory response in the periodontal tissues, resulting in structural damage. Vasodilation and increased vascular permeability cause tissue swelling and erythema, which lead to the deepening of periodontal pockets. Inflammatory cells, particularly neutrophils, infiltrate the tissues, causing collagen degradation beneath the epithelial layer. The epithelial tissue responds by proliferating in an effort to maintain tissue integrity. However, the degeneration of fibroblasts inhibits tissue repair, leading to apical migration of the epithelial cells, further exacerbating the periodontal pocket. The subgingival bacteria then invade these pockets, and as the epithelium lacks its own blood supply, cells farther from the connective tissue experience necrosis, contributing to the formation of the periodontal pocket [1,3,[50], [51], [52]].

In this study, the group of rats treated with *Peperomia pellucida* extract showed a significant reduction in probing pocket depth (PPD) on days 5 and 7 compared to the untreated periodontitis-induced group. This finding is consistent with previous studies using curcumin and baicalein, where a reduction in probing depth and an increase in alveolar bone were observed [53,54]. Moreover, there were no significant differences between the *Peperomia pellucida* group and the normal or positive control group treated with 0.2 % chlorhexidine (CHX). These results suggest that *Peperomia pellucida* promotes tissue attachment and enhances healing in periodontitis-induced rats. The chromatography results in this study indicate that *Peperomia pellucida* contains flavonoids and polyphenols These compounds inhibit signaling pathways such as NF-κB and MAPK, which are involved in the regulation of inflammatory responses, oxidative stress, and cellular repair [25,26]. Compared to other flavonoid-rich extracts, such as green tea and turmeric, *Peperomia pellucida* offers similar anti-inflammatory benefits [55,56]. However, the concentration and types of flavonoids may vary, which could influence the overall efficacy. Further comparative studies would be required to understand the full scope of these differences.

Chlorhexidine is well-recognized for its potent antibacterial effects within periodontal pockets and is considered one of the most effective adjunctive treatments for periodontitis. Its mechanism of action involves the attraction of cationic chlorhexidine molecules to the negatively charged bacterial cell surfaces, leading to bacterial cell death. Similarly, *Peperomia pellucida* is rich in flavonoids, which have been extensively studied for their anti-inflammatory and antimicrobial properties [[57], [58], [59], [60]]. Flavonoids are known to inhibit the production of reactive oxygen species (ROS) and proinflammatory cytokines, both of which play key roles in periodontal inflammation. Additionally, flavonoids have been found to promote the proliferation of fibroblasts, which are essential for tissue regeneration and repair, leading to reduced PPD and bleeding upon probing [61].

The antibacterial properties of both chlorhexidine and Pepper Elder extract help create a cleaner oral environment, thereby facilitating fibroblast proliferation. Studies have demonstrated that chlorhexidine and Pepper Elder extract can enhance fibroblast cell growth, which contributes to faster wound healing. For example, in a previous study involving Wistar rats, mucosal wounds treated with 0.2 % chlorhexidine showed a significant increase in fibroblast counts, leading to accelerated healing [37,62]. Another study on burn-induced wounds in rats treated with betel leaf extract, which contains similar flavonoids, also reported enhanced fibroblast activity, further supporting the regenerative potential of these compounds [63].

The serum levels of TNF-α and IL-1β, key proinflammatory cytokines, were highest in the group of rats induced with periodontitis without treatment, confirming the presence of active inflammation. However, in the group treated with Pepper Elder extract, these cytokine levels were significantly lower, approaching the levels seen in the pristine group, and were comparable to those in the chlorhexidine-treated group. This suggests that Pepper Elder extract effectively reduces the levels of TNF-α and IL-1β, mitigating tissue damage and alveolar bone loss, which are hallmark features of periodontitis [3,12,53,64].

Herbal treatments are increasingly explored for their role in periodontal therapy, particularly their effects on cytokine modulation. For instance, studies on green tea extract (*Camellia sinensis*) have demonstrated its ability to reduce levels of proinflammatory cytokines such as TNF-α and IL-1β in periodontitis models. In one study, green tea polyphenols effectively reduced periodontal inflammation and alveolar bone loss through downregulation of these cytokines, indicating similar anti-inflammatory and antimicrobial properties to *Peperomia pellucida*. Additionally, a study on *Curcuma longa* (turmeric) revealed its efficacy in reducing TNF-α levels and inhibiting matrix metalloproteinase (MMP) activity, which plays a critical role in tissue destruction during periodontitis. These findings are consistent with the outcomes of our study, which showed that *Peperomia pellucida* extract significantly reduces TNF-α and IL-1β levels, comparable to CHX treatment [55,56]. Flavonoids are known to have anti-inflammatory and antioxidant properties, and some flavonoids have shown inhibitory effects on MMPs in other studies [26]. Exploring this potential in *Peperomia pellucida* could provide additional therapeutic benefits by preventing further tissue degradation and promoting periodontal health.

Anti-inflammatory cytokines such as IL-10 and IL-13 play a crucial role in regulating the balance between inflammation and tissue healing. In this study, IL-10 levels were significantly higher in the group treated with *Peperomia pellucida* extract, similar to the chlorhexidine group. IL-13 levels also showed a significant difference compared to the negative control group, while no difference was observed when compared to the pristine and CHX 0.2 % treatment groups. These findings suggest that *Peperomia pellucida* enhances anti-inflammatory responses, particularly through the regulation of IL-10 and IL-13, both of which contribute to modulating inflammation in periodontitis. In a related study on *Azadirachta indica* (neem), the extract was found to increase IL-10 production, leading to improved clinical outcomes in periodontitis-induced animal models. Similarly, *Allium sativum* (garlic) extract was shown to elevate IL-10 and IL-13 levels, providing protective effects against alveolar bone loss and promoting anti-inflammatory responses in periodontal disease. These cytokines help suppress proinflammatory signals, promote tissue regeneration, and inhibit the activity of osteoclasts, thereby preventing further bone loss [10,49,[65], [66], [67], [68]]. Future research could further investigate its role in sustaining periodontal health and preventing disease recurrence.

The primary focus in this study was on evaluating inflammatory markers, such as TNF-α, IL-1β, IL-10 and IL-13, to determine the anti-inflammatory effects of the extract. Future studies may need to explore whether the extract also affects the bacterial load in the periodontal pockets, impact on vascular changes such as vasodilatation or permeability to provide a more comprehensive understanding of its potential therapeutic benefits in periodontitis management. A more extended follow-up could provide valuable insights into the sustainability of the treatment's effects, including whether the benefits observed in the short term continue to improve or maintain tissue and bone health over time. This would help in understanding the full therapeutic potential of Peperomia pellucida for long-term periodontal health management. There is potential evidence suggesting that Peperomia pellucida extract could be combined with other herbal or pharmaceutical treatments to enhance its efficacy in periodontal therapy. Given its anti-inflammatory properties, it may complement other agents, such as chlorhexidine or plant-based treatments like green tea or turmeric, which target different pathways, such as antimicrobial activity or antioxidant effects. Combining these treatments could provide a more holistic approach to managing periodontitis, addressing both bacterial load and inflammatory responses more effectively. Further research would be needed to confirm this synergistic potential.

The findings of this study could significantly inform the development of other plant-based treatments for periodontitis by highlighting the therapeutic potential of targeting specific cytokine profiles and signaling pathways. *Peperomia pellucida's* ability to modulate both pro-inflammatory and anti-inflammatory cytokines suggests that other plants with similar bioactive compounds could be explored. Additionally, these results may encourage researchers to investigate plant extracts that influence other molecular pathways, such as oxidative stress reduction or tissue regeneration, leading to more comprehensive treatment options.

## 5 Conclusion

This study demonstrates the potential of Pepper Elder extract as an effective alternative treatment for periodontitis, owing to its anti-inflammatory and antimicrobial properties. While chlorhexidine remains a gold standard for periodontal therapy, Pepper Elder extract offers a natural, plant-based option with comparable benefits in reducing periodontal inflammation and supporting tissue healing. Further research is necessary to explore its long-term efficacy and potential applications in clinical settings.

## Author contributions

Dewi Lidya Ichwana Nasution: Conceptualization, methodology, Data Analysis, Writing-Original draft. Sri Tjahajawati: Investigation, Resources, Writing-Review and Editing. Ratna Indriyanti: Investigation, Resources, Writing-Review and Editing. Amaliya: Investigation, Resources, Writing-Review and Editing.

## Declaration of competing interest

We wish to confirm our manuscript with the title below:

There are no known conflicts of interest associated with this publication and there has been no significant financial support for this work that could have influenced its outcome. We declare that **Dewi Lidya Ichwana** as an author has no financial or personal relationships with other people or organizations that could inappropriately influence or bias the content of this paper.

## Data Availability

I have provided data alongside the article
